# Evaluation of Corrosion Damage in Sulfate-Attacked Concrete by CT, Ultrasonic Pulse Velocity Testing and AHP Methods

**DOI:** 10.3390/s22083037

**Published:** 2022-04-15

**Authors:** Dunwen Liu, Chun Gong, Yu Tang, Yinghua Jian, Kunpeng Cao, Haofei Chen

**Affiliations:** School of Resources and Safety Engineering, Central South University, Changsha 410083, China; dunwen@csu.edu.cn (D.L.); tangyu12@csu.edu.cn (Y.T.); jyh__0412@csu.edu.cn (Y.J.); 205511007@csu.edu.cn (K.C.); 205512072@csu.edu.cn (H.C.)

**Keywords:** corrosion evaluation, sulfate attack, ultrasonic pulse velocity, coarse aggregate, CT test, AHP

## Abstract

Coarse aggregate in concrete is basically free from sulfate corrosion. If the influence of the coarse aggregate in the concrete is not eliminated, the change amount of the concrete ultrasonic pulse velocity value is directly used to evaluate the damage degree of sulfate corrosion in the concrete, and the results are often inaccurate. This paper presents an evaluation method of corrosion damage for the sulfate-attacked concrete by CT, ultrasonic velocity testing and AHP methods. CT was used to extract the coarse aggregate information in the specimen, and the proportion of coarse aggregate on the ultrasonic test line was calculated based on CT image analysis. Then, the correction value of ultrasonic pulse velocity (UPV) of the concrete structure was calculated, and the sulfate corrosion degree of concrete structure was evaluated using the analytic hierarchy process (AHP). The results show that the evaluation method proposed in this paper could more accurately evaluate the corrosion damage in the sulfate-attacked concrete structures, and the evaluation results were more in line with reality.

## 1. Introduction

The durability of concrete is very important to the service life of civil infrastructure, and sulfate erosion is one of the most critical factors affecting the durability of concrete structures [1,2]. Some buildings, such as underground engineering, tunnels and offshore concrete structures, are in a sulfate corrosive environment [3,4,5]. Sulfates are naturally occurring minerals found in saline–alkali soil, groundwater and coastal areas [6]. Salt lakes and saline soils in western and coastal areas of China are rich in sulfate aggressive ions (i.e., sulfate, chloride and magnesium) [7]. The external sulfate corrosion environment will affect the performance of the concrete structure and reduce its safety [6,8]. Therefore, it is necessary to diagnose the state of existing buildings through several detection methods to ensure their safety and durability and prevent their failure. At present, there are many methods to evaluate the performance of concrete structures, mainly by detecting factors such as internal cracks [9,10], compressive strength [11], porosity of the concrete and combining several analysis methods. Some researchers have also proposed new assessment methods and indicators. Hanbin Cheng [12] et al. defined an overall distribution area of sulfate ions to describe the nonuniform degradation behavior of sulfate-attacked concrete and used it to predict the degradation level of concrete structures under sulfate corrosion. According to the literature [9,10,11,12], many methods currently require damage experiments on concrete structures to test the sulfate content inside the concrete structure, which results in a more complicated experiment process. However, using non-destructive testing may have the problem of lower test accuracy or higher cost. Therefore, this article intends to propose a corrosion assessment of sulfate-attacked concrete with simple operation, lower cost and higher accuracy.

At present, a large number of studies have been carried out on the mechanism of sulfate attack. The results generally show that sulfate attack is mainly divided into two categories: chemical corrosion and physical (crystalline) corrosion [13,14]. Chemical corrosion is usually caused by the reaction between the sulfate that has invaded the concrete structure and the components in the Portland cement to produce ettringite, gypsum, magnesium sulfate, moissanite, etc. The chemical corrosion process of sulfate causes the cement matrix composition and the PH value inside the concrete structure changes [13,14,15]. The physical attack process is the supersaturation of the external sulfate solution to produce crystallization and the increase in the volume of the product during the chemical corrosion process leads to an increase in expansion stress, which leads to cracking and failure of the concrete structure [13,14,15]. As the mechanism of sulfate attack is very complicated, there are many factors affecting the corrosion process. The kind of cation in sulfates also has a great influence on the chemical process [16]. For example, magnesium ions combine with hydroxide to form brucite, which reduces the alkalinity of pore solution and decomposes cement hydration products such as calcium–silicate–hydrate (C-S-H) gel [13]. It can be seen from the sulfate corrosion mechanism that sulfate only attacks the cement matrix in the concrete structure but does not attack the coarse aggregate.

As an important material in infrastructure construction, concrete has been widely used in structures such as house construction, highway tunnels, and bridges [17]. In the actual operation of concrete buildings (structures), due to the influence of some harsh environments, there will be degradation and corrosion phenomena for different reasons. Test methods are usually used to detect the corrosion of concrete, and the corresponding intervention measures are determined according to the test results so as to extend the service life of concrete buildings (or structures). For large buildings, destructive testing will affect the performance of the building structure; therefore, there have been an increasing number of projects that use non-destructive testing (NDT) to evaluate the durability of concrete structures. NDT refers to the detection of immediate internal damage or defects in concrete structures without destroying the concrete structure. In actual work, we must not only discover the defects of the concrete structure but also estimate whether the structure can continue to be used. Therefore, it is necessary to evaluate the overall corrosion of concrete structures. Non-destructive technology based on the propagation of elastic waves is well known and has successfully been applied to the inspection of building materials such as concrete [18]. Ultrasonic testing (UT) is one of the most commonly used non-destructive testing technologies due to the fact of its simplicity of operation; it can easily be used at construction sites and provides results promptly [19,20,21,22]. They are based on the fact that the wave propagation velocities (WPVs) depend on the density and the stiffness of the material (Young’s modulus and Poisson coefficient for linear elastic isotropic materials) [18]. For heterogeneous materials, such as concrete, the relationship between the mechanical properties of the material and the speed of ultrasonic sound can be derived based on the elastic wave theory, and data on the various conditions need to be used for correction, because they may be affected by factors such as aggregate and water content [23,24,25]. As the compactness of concrete structures will change under sulfate attack, we can reflect the sulfate corrosion of concrete structures through ultrasonic pulse velocity (UPV). UPV is often used to detect relatively homogeneous materials such as mortar and rock [21,26]. Concrete is a heterogeneous material. The internal coarse aggregate is unevenly distributed and is not affected by sulfate corrosion, which has a great influence on the overall UPV of the concrete structure, and the complexity of it makes the ultrasonic waves in concrete highly irregular [27]. If we can ensure the proportion of the coarse aggregate inside the concrete structure on the ultrasonic survey line and calculate the UPV of the non-coarse aggregate part of the concrete, it will be more precise to use the change in the UPV of the non-coarse aggregate inside the concrete to estimate the degree of sulfate corrosion of concrete structures.

To confirm the proportion of coarse aggregate in a concrete structure, it is necessary to obtain an image of the internal structure of the concrete. Computed tomography (CT), as a non-destructive testing method, is widely used to explore the internal structure of concrete and its development [28,29,30,31]. It can clearly, accurately and intuitively display the internal structure, composition, material and defect status of the detected object in the form of two-dimensional tomographic images or three-dimensional images under the condition of no damage to the detected object [32,33,34], and it is known as the best non-destructive testing and non-destructive assessment technology today. Therefore, CT makes it possible to ensure the proportion of the coarse aggregate inside the concrete structure on the ultrasonic survey line so as to use the change in the UPV of the non-coarse aggregate part to more accurately judge the sulfate corrosion degree of the concrete structure. The only disadvantage of CT is that the cost of testing is too high. Since the coarse aggregate is not attacked by the sulfate inside the concrete structure, it only needs to be tested once with CT, which greatly saves on the cost of CT.

In addition, the distribution of coarse aggregates in the concrete structure is not uniform, and the UPV of a survey line cannot reflect the overall corrosion degree of the concrete structure. Therefore, in this article we evaluated the overall corrosion of concrete structures through UPV in multiple directions. In the process of concrete corrosion, the more severe the corrosion, the greater the impact on the overall performance of the concrete structure. By assigning weights to the UPV in different test directions, the overall corrosion situation of the concrete structure can be more accurately reflected. AHP, being an expert-driven data mining technique, was introduced by Saaty [35]; it is usually used for semi-quantitative research of qualitative indicators [36], and it determines the judgment matrix by means of expert scoring, which makes the calculation result more subjective. In this article, AHP was used to calculate the weights of UPV in various directions, and the judgment matrix was determined by the ratio between each direction. For the first time, specific values were used to determine the judgment matrix, which eliminated the influence of subjective factors and made the calculation results more accurate.

## 2. Materials and Methods

### 2.1. Specimen Preparation and Experimental Design

**Figure 1:**
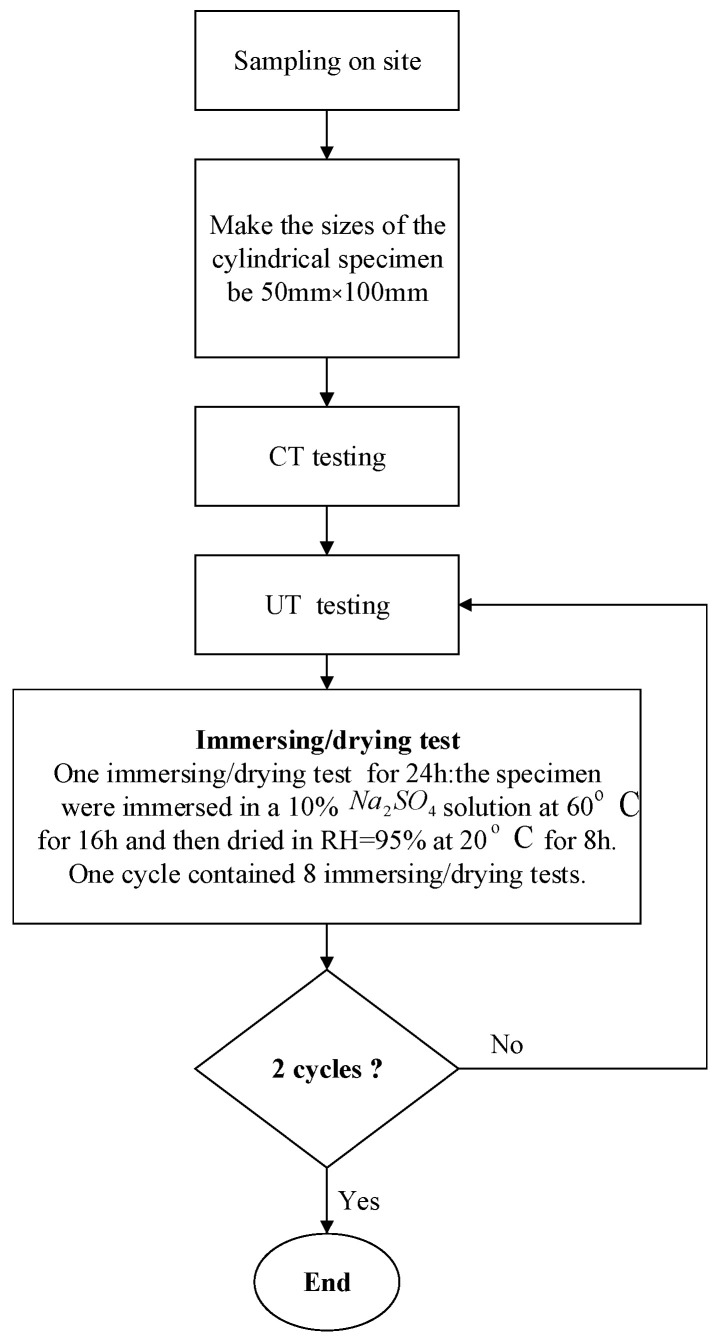
Experimental steps.

In this study, the samples were taken from the corroded section of a highway tunnel in Chongqing, China. The specific experimental steps are shown in Figure 1.

The concrete blocks were made into 50 × 100 mm cylindrical samples for dry–wet cycle accelerated corrosion experiments. In the experiments in this paper, each immersing/dry cycle was 24 h (i.e., one day). Every eight days was a cycle, and this experiment was carried out for two cycles. The experimental samples were tested by ultrasonic and CT before the experiment and after the end of each cycle. In theory, the proportion of coarse aggregate on the ultrasonic measurement line can be obtained by only one CT scan test of the sample. In order to determine the accuracy of the evaluation method proposed in this article, we performed CT on the specimen after each dry–wet cycle and calculated its porosity changes.

### 2.2. Testing Devices and Methods

#### 2.2.1. Ultrasonic Pulse Velocity Testing (UPV)

UPV is a non-destructive testing method to effectively measure the quality of concrete. It is a function of the square root of the ratio of dynamic elastic modulus (*E*) and its density (ρ) [37].

(1) $$V=f{\left(\frac{gE}{\rho }\right)}^{1/2}$$

where *g* is the acceleration due to the fact of gravity, m/s^2^.

In the same material, the strength is directly proportional to the dynamic modulus of elasticity. It is the detection principle of ultrasonic testing to indirectly reflect the strength of the material through the relationship between the ultrasonic pulse velocity and the dynamic elastic modulus. Generally speaking, the smaller porosity, the denser the material and the larger the UPV. In general research, the UPV in one direction of the concrete structure is often used to reflect the overall performance of the concrete structure, but the concrete structure is a heterogeneous material, and the uneven distribution of coarse aggregates will cause differences in the strength of each part of the concrete structure. Therefore, only one direction of the UPV in the concrete structure cannot reflect the overall corrosion of the concrete structure. Therefore, in this paper, we propose to measure UPV from multiple directions of concrete structures and establish a system for calculating the overall corrosion degree of concrete structures through AHP.

**Figure 2:**
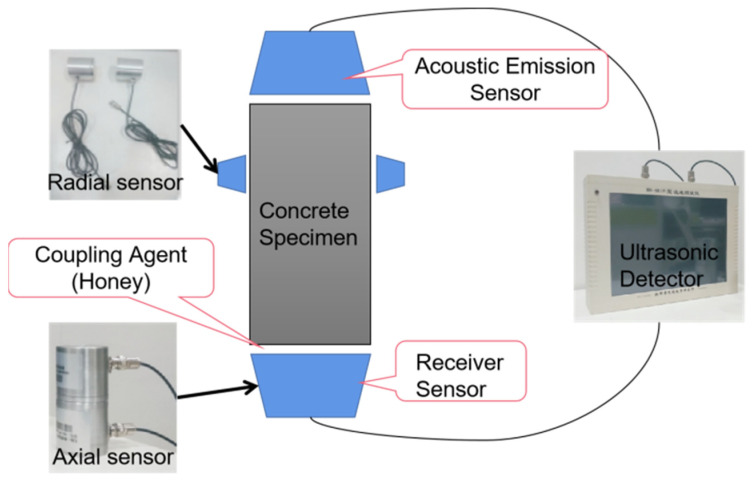
Ultrasonic testing system.

**Figure 3:**
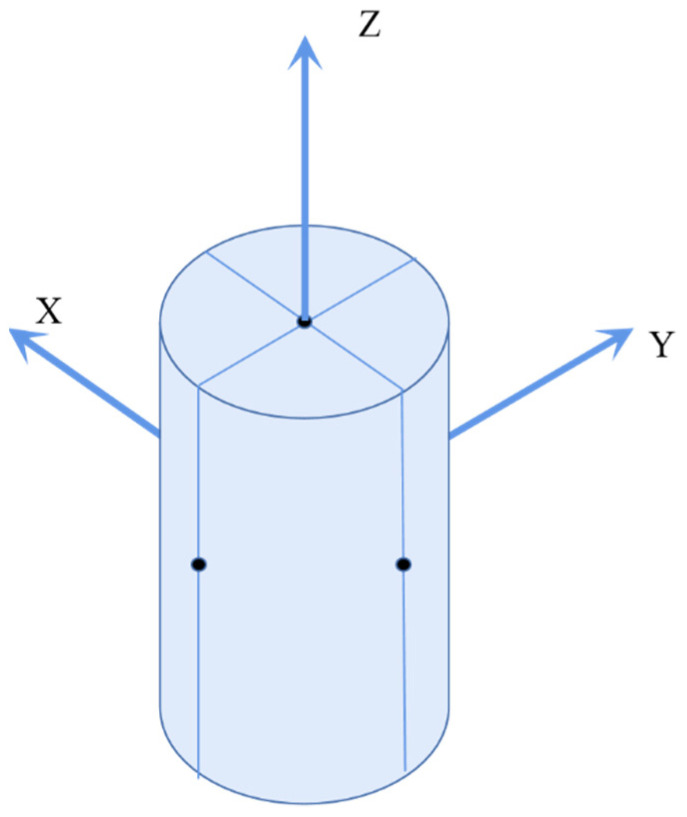
Distribution of measurement.

In this paper, the UPV of the concrete structure was measured by an ultrasonic detector. We use a customized HS-CS1H ultrasonic detector in which the main frequency of the instrument was 20 MHz and the frequency of the transducer was 1 MHz; the ultrasonic testing system is shown in the Figure 2. The ultrasonic detector was composed of two sensors and a host computer. The sensors, which were used to measure the axial longitudinal wave velocity and the radial longitudinal wave velocity of the concrete structure, were different. In ultrasonic testing, if the distance between the transducers is much larger than the wavelength of the ultrasonic waves, it can be considered that the mechanical wave propagates in a straight line between the transmitter and receiver transducers [38]. Therefore, we should measure the UPV between a couple of points. In principle, the more points measured, the more accurate the overall situation of the concrete will be reflected. But taking into account the size of the concrete sample used in this article and the size of the sensors, we measured the UPV of a concrete specimen in three directions with 6 points to calculate the relative corrosion degree of the sample as shown in the Figure 3.

#### 2.2.2. X-ray CT Technique

The X-ray CT technique can accurately and intuitively show the internal structure, material and defects of the measured object in the form of two-dimensional tomographic images or three-dimensional images. It is a very accurate non-destructive testing method.

**Figure 4:**
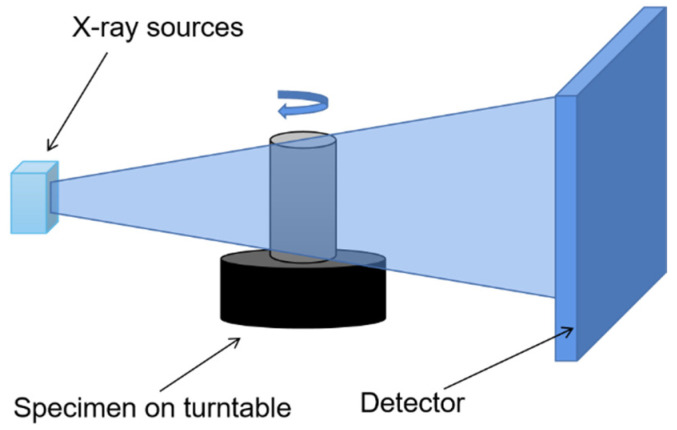
225 KV industrial CT.

**Figure 5:**
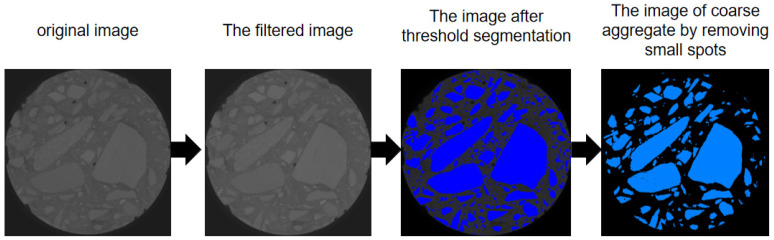
The processing steps of the CT images.

In this paper, every specimen was imaged using a 225 KV industrial CT scanner (see Figure 4). The concrete specimen was imaged, which resulted in volumes of 2048 × 2048 × 2048 voxels, with a voxel size of 62.251 μm. The Avizo software was used to edit the original images, and the processing steps are shown in Figure 5. First, we filtered the original image to reduce the artifacts generated in the CT detection process. Then, we used threshold segmentation to roughly segment the coarse aggregate of concrete and, finally, removed the small spots after segmentation to obtain the distribution image of coarse aggregate.

### 2.3. Sulfate Corrosion Assessment Based on AHP

#### 2.3.1. Overview of the Method

The AHP is a theory of measurement through pairwise comparisons and depends on expert opinions to derive the priority [39]. Therefore, the results of the evaluation are easily affected by the subjective consciousness of experts. In this paper, AHP was used in data processing, and an AHP-based evaluation model for the relative corrosion coefficient of concrete was established. The judgment matrix was established by comparing the ultrasonic velocities in all directions, and there was no subjective factor affecting the establishment of the judgment matrix, which made the evaluation results more objective. In this article, In this paper, three directions of concrete samples were tested, and the overall steps are as follows.

Step 1: The specimens by the drill core were tested for longitudinal wave velocity measurement from the directions of X, Y and Z. Then, we calculated the UPV of the overall concrete using Equation (2):

(2) $$V_{i}=w_{x_{i}}V_{X_{i}}+w_{y_{i}}V_{Y_{i}}+w_{z_{i}}V_{Z_{i}}$$

In the equation, $V_{i}$ is the overall UPV of the concrete structure after the *i*-th dry–wet cycle experiment. $w_{x_{i}}$ ($w_{y_{i}}$ or $w_{z_{i}}$) is the weight of the UPV in the X (Y or Z) direction of the concrete specimen after the *i*-th dry–wet cycle experiment. $V_{x_{i}}$ ($V_{y_{i}}$ or $V_{z_{i}}$) is the UPV of the cores drilling into the specimens in the X (Y or Z) direction after the i-th dry–wet cycle experiment.

Step 2: Then, we calculated the relative corrosion coefficient of concrete using Equation (3).

(3) $$K_{i}={\left(\frac{V_{i}}{V_{0}}\right)}^{{}^{{}_{2}}}$$

In the equation, K is the relative corrosion coefficient of concrete; $V_{i}$ is the same as above; $V_{0}$ is the UPV of the concrete specimen before the experiment.

The above evaluation method for the corrosion degree of concrete components can more comprehensively consider the corrosion degree of concrete in all directions so as to calculate the relative corrosion coefficient of concrete components more completely.

#### 2.3.2. Establishment of the Comprehensive Evaluation Model Based on AHP

In this part, we explain the calculation process of the concrete relative corrosion coefficient in detail with AHP.

The UPV in the medium reflects the properties of the material indirectly. With the development of material damage, the longitudinal wave speed will also decrease [40]. Next, we defined the damage index (DI) as C, and it was calculated according to Equation (4).

(4) $$C_{jk}=\frac{V_{j}}{V_{\begin{array}{l} k \end{array}}}$$

In the equation, j (or k) is x, y or z.

According to the value of C, the judgment value of the relative concrete corrosion degree was obtained according to Table 1, and the judgment matrix of the test 3D judgment matrix is shown in Table 2.

In addition, in the table $V_{jk}=\frac{1}{V_{kj}}$.

We can obtain the judgment matrix (Equation (5)) according to Table 2.

(5) $$V=\left[\begin{array}{ccc} V_{11} & V_{12} & V_{13}\\ V_{21} & V_{22} & V_{23}\\ V_{31} & V_{32} & V_{33} \end{array}\right]$$

We can approximate the value of the evaluation factor weight vector with the following Equation (6):

(6) $$w_{i}{}^{\prime }=(\prod_{j=1}^{3}V_{ij}{)}^{\frac{1}{3}}(i=1,2,3)$$

Calculation of the normalized weight can be conducted using Equation (7):

(7) $$w_{i}=\frac{w_{i}{}^{\prime }}{\sum_{i=1}^{3}w_{i}{}^{\prime }}$$

The accuracy of pairwise comparisons was calculated through the consistency index (CI). However, the consistency of judgement was checked by calculating the consistency ratio (CR). The CR controls the balance in which weights are assigned [41]. The acceptable CR level is <0.10. Equation (8) for CR is:

(8) $$CR=\frac{CI}{RI},$$

Because this is a three-factorial matrix, the CI for a matrix was calculated using the following Equation (9):

(9) $$CI=\frac{\lambda_{\max}-3}{3-1},$$

According to the random index (RI) table (Table 3), the RI is 0.52.

Finally, the AHP was used to obtain the weight value of each direction and then put into Equation (3) to obtain the relative concrete corrosion coefficient.

#### 2.3.3. Analysis of the Concrete Corrosion Classes

After obtaining the concrete corrosion coefficient, we put forward a set of evaluation classifications of the concrete corrosion degree. First, we defined the absolute damage index (ADI) as per Equation (10):

(10) $$C^{\prime }=\min\left\{\frac{V_{x}}{V_{0}},\frac{V_{y}}{V_{0}},\frac{V_{z}}{V_{0}}\right\}$$

The evaluation classification of the relative corrosion degree is shown in Table 4.

## 3. Result and Discussion

### 3.1. Analysis of Concrete Coarse Aggregate Based on CT

**Figure 6:**
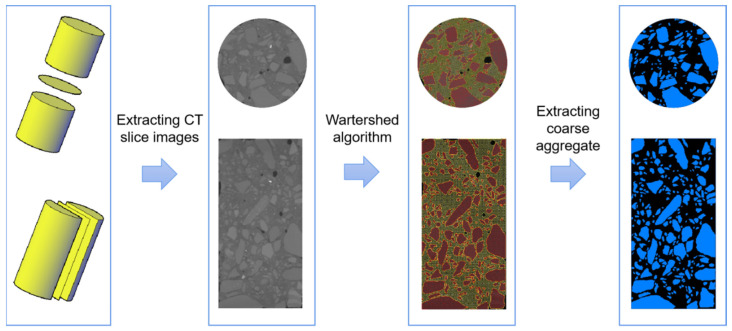
The extraction process of the coarse aggregate information corresponding to the ultrasonic survey line of the CT image.

The coarse aggregate of a concrete structure will not change under sulfate attack. Therefore, we only needed to perform a CT scan on the specimen and then obtain the coarse aggregate information corresponding to the ultrasonic survey line and extract it; the specific process is shown in Figure 6.

In this article, we took three-directions of the UPV of the specimen for measurement; thus, only three-directional coarse aggregate information needed to be extracted from the CT images for analysis. First, we extracted the slice corresponding to the location of the ultrasonic survey line, and then used the watershed algorithm to segment the pores, cement and coarse aggregate of the concrete and, finally, extracted the slice image containing only the coarse aggregate.

### 3.2. Correction Calculation of the UPV of the Concrete Structure

**Figure 7:**
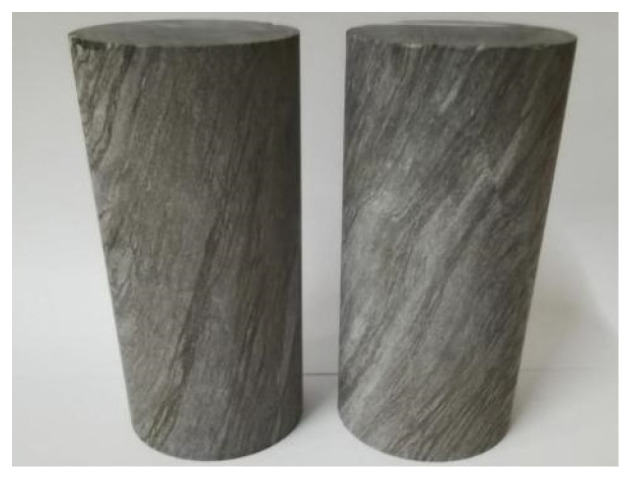
Limestone.

The coarse aggregate in the specimen of this article was limestone (Limestone) material. In order to remove the impact of coarse aggregate on the overall sound velocity of the specimen, we collected limestone from the site with the same material as the coarse aggregate in the specimen tested this time and formed them into two cylindrical samples of 50 × 50 mm as shown in Figure 7. Their UPV values are shown in Table 5, and the average value was taken as the UPV of the coarse aggregate.

**Figure 8:**
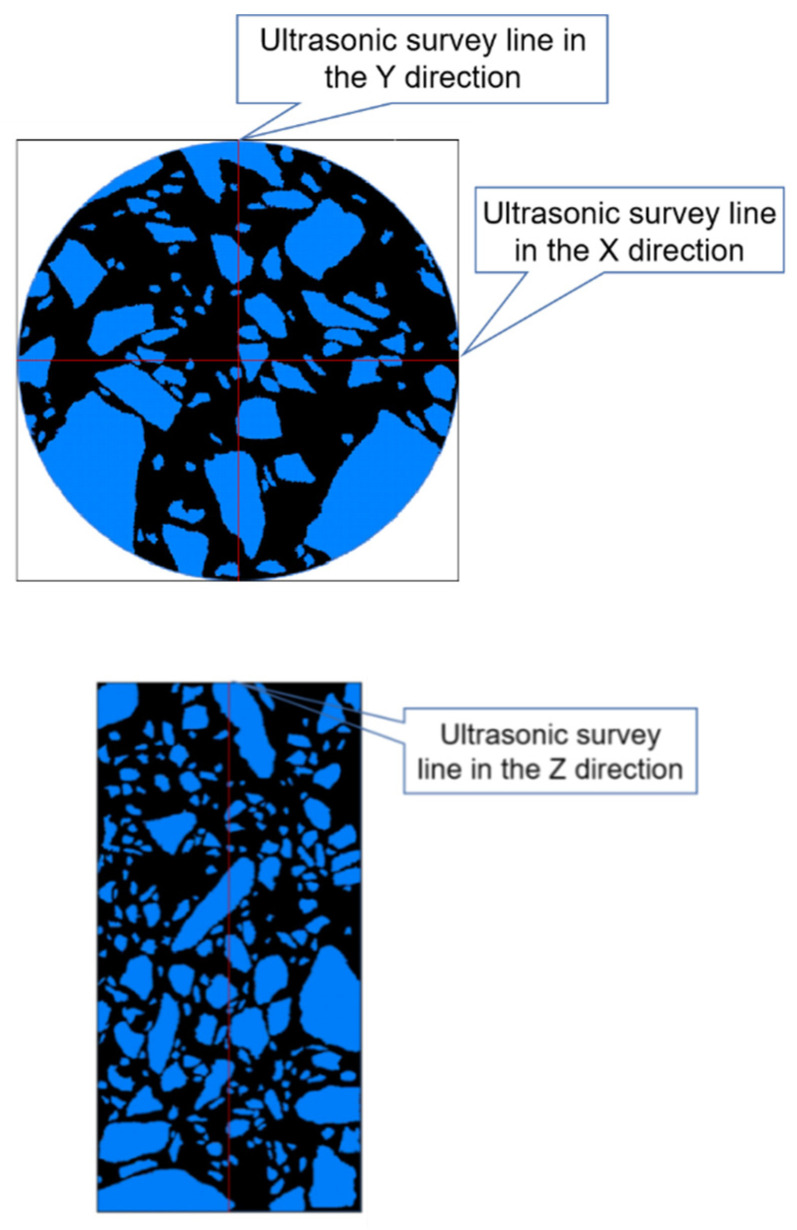
Correspondence between the ultrasonic survey line and the CT image.

The correspondence between the ultrasonic survey line and the extracted coarse aggregate slices is shown in Figure 8, and the length of the coarse aggregate on the ultrasonic survey line was marked with CAD software.

**Figure 9:**
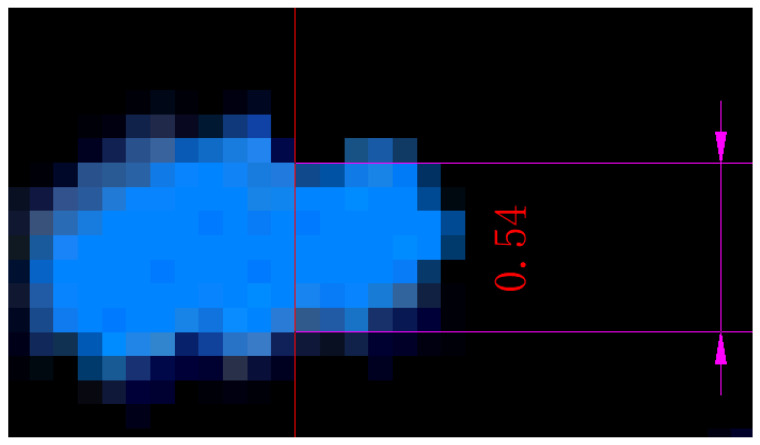
Schematic diagram of the marking the length of coarse aggregate.

**Figure 10:**
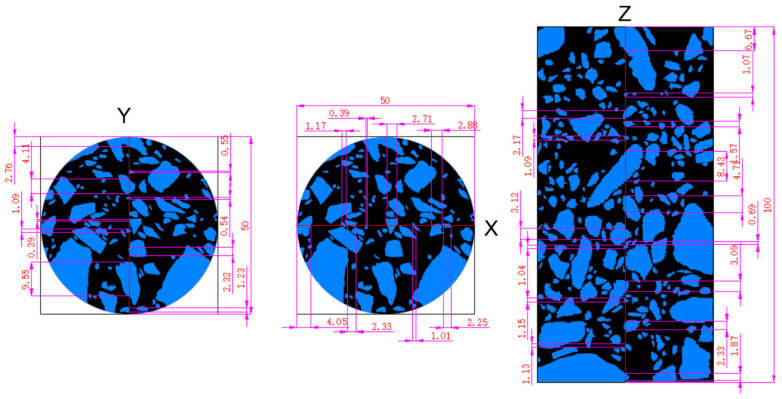
Schematic diagram of marking the length of coarse aggregate on ultrasonic measuring line (mm).

We used CAD software to mark the length of the coarse aggregate that the survey line passed through. The specific marking method is shown in Figure 9. By enlarging the image in CAD to select the two endpoints of the ultrasonic survey line passing through the coarse aggregate for marking, the results of marking the length are shown in Figure 10.

The proportion of coarse aggregate on the ultrasonic survey line was obtained as shown in Table 6

According to Equation (11), the corrected values for UPV without the effect of coarse aggregate are shown in Table 7.

(11) $$V_{Corrected\;value}=\frac{1-\omega }{\frac{1}{V_{Measuring\;value}}-\frac{\omega }{V_{Coarse\;aggregate}}},$$

### 3.3. The Results of the Corrosion Assessment of Sulfate-Attacked Concrete Based on AHP

The specimen in this article was taken from a corroded section of a highway tunnel in Chongqing, China, which has experienced severe sulfate corrosion. Therefore, through the accelerated indoor test, the corrosion rate of the specimen used in this paper was faster than the new concrete specimen.

**Figure 11:**
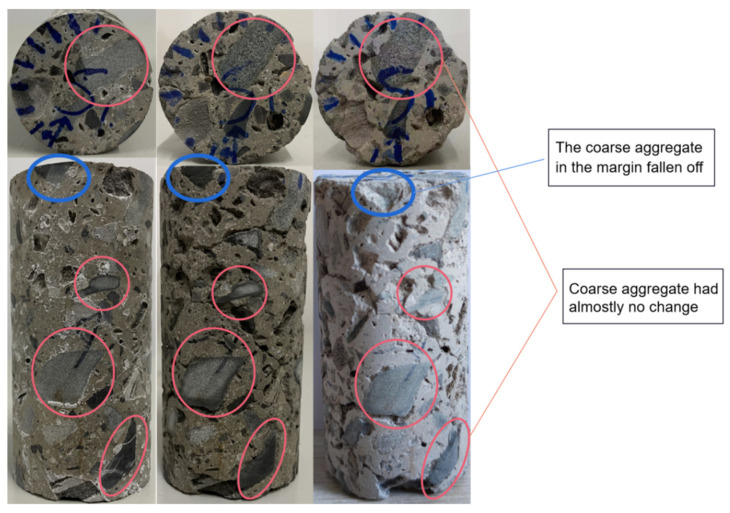
Appearance changes of the concrete specimen.

In Figure 11, after two sulphate dry–wet cycle experiments, the appearance of the concrete specimens changed significantly, and a large amount of cement matrix on the surface fell off, but almost all of the coarse aggregates did not change. Only one part of the coarse aggregate fell off at the edge, which was caused during the cutting process of the specimen. In addition, the measuring point in this experiment did not pass through the drop zone of this aggregate, which did not affect the calculation results of the experiment. At the same time, it can be seen from Figure 11 that the degree of corrosion of the concrete specimens in all directions was extremely different. Therefore, it was necessary to evaluate the overall corrosion of the concrete samples by testing UPV in multiple directions.

Taking into account the heterogeneity of the concrete materials, the AHP-based corrosion assessment method of concrete structure proposed in this paper was used to evaluate the sulfate corrosion of the samples. First, we calculated the corrected UPV values for each experimental state according to Equation (2). The specific values are shown in Table 8. Then, the UPV of the concrete specimen before the start of the experiment was taken as the initial velocity, and the sulfate relative corrosion coefficient of the specimen after two wet–dry cycle experiments was calculated. The results were shown in Table 9.

### 3.4. Evaluation Result Verification

#### 3.4.1. Change in Appearance of Concrete Specimen

It can be seen from relative corrosion coefficient results that the concrete structure calculated by the measuring value were all mild corrosion, the range of changes was small. However, from the apparent change in the concrete (see Figure 11), it can be seen that the surface of the concrete sample underwent significant corrosion changes after two dry and wet cycles; the relative corrosion coefficient calculated by corrected value was more in line with the actual situation. Through non-destructive testing experiments, it is found that the UPV of coarse aggregate and its proportion were large, and slight changes in the cement matrix and pores were difficult to reflect from the overall ultrasonic velocity. The corrected UPV, excluding the influence of coarse aggregate, reflects the actual changes in sulfate corrosion; therefore, the evaluation results based on the corrected UPV were more accurate.

#### 3.4.2. Change in the Porosity of the Concrete Specimen

**Figure 12:**
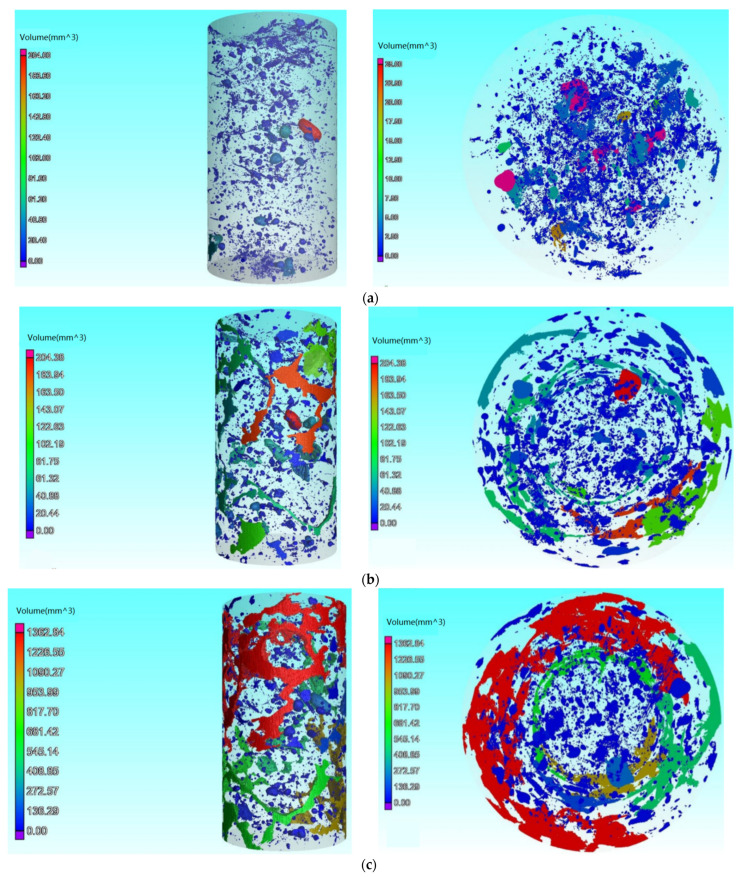
Three-dimensional pore distribution in the concrete specimen. (**a**) Pore distribution in the concrete specimen in its original state. (**b**) Pore distribution in the concrete specimen after the first circle. (**c**) Pore distribution in the concrete specimen after the second circle.

In order to verify the correctness of the evaluation method proposed in this paper, we carried out CT scanning tests on the concrete specimen at each experimental stage and used the scanning results to reconstruct the pores of the concrete specimen in three dimensions as shown in Figure 12.

Judging from the three-time pore structure distribution of the concrete samples in Figure 12, the pores of the concrete samples increased significantly after two accelerated dry–wet cycle experiments, indicating that the compactness of the concrete samples were significantly reduced; thus, the relative corrosion coefficient of the concrete sample should be significantly reduced.

**Figure 13:**
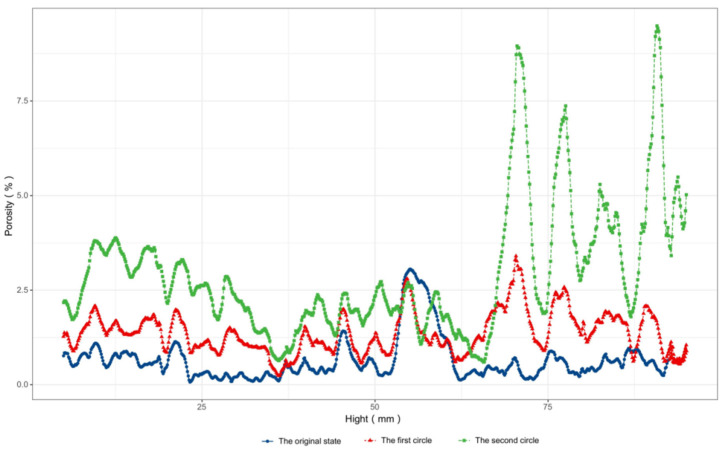
Concrete sample porosity vertical height distribution.

In this paper, the porosity of a three-dimensional reconstructed model was calculated, and the change in the porosity of the concrete with the height of the concrete sample was obtained as shown in the Figure 13.

The porosity of the three-dimensional reconstructed model was calculated, and the change in the porosity of the concrete with the height of the concrete sample was obtained as shown in Figure 8. It can be seen from the figure that the corrosion of each height of the concrete sample was extremely uneven, and the porosity of some areas was reduced during the corrosion process, because the product formed by the reaction of external sulfate and the cement matrix filled the original pores’ structure. With the increase in sulfate corrosion products, expansion stress inside the concrete sample caused part of the cement matrix to crack or even fall off, which made the overall porosity increase. The calculation results of the overall porosity of the concrete specimen are shown in Table 10. It can be seen that the porosity of the concrete sample showed an obvious upward trend, which is consistent with the result obtained using the corrected values of UPV, indicating that the overall relative corrosivity evaluation result of the concrete structure calculated by the corrected values of the UPV was more accurate.

## 4. Conclusions

This paper proposed an evaluation method for corrosion damage in sulfate-attacked concrete based on the CT, ultrasonic pulse velocity testing and AHP. CT was used to extract the coarse aggregate information in the specimen, and the proportion of coarse aggregate on the ultrasonic test line is calculated. Then, the corrected UPV values of concrete structure were calculated, and the degree of sulfate corrosion of the concrete structure was evaluated combined with the analytic hierarchy process. The main conclusions are as follows:

(1) A CT-based analysis method for the proportion of the length of the coarse aggregate in concrete along an ultrasonic survey line was proposed. The internal structural characteristics of concrete can be accurately extracted using CT scanning, and the distribution and size of the coarse aggregate in the concrete specimen were obtained using Avizo image processing software and determined the proportion of coarse aggregate length on a certain ultrasonic test line;
(2) A method for analyzing the influence of removing coarse aggregates in concrete structures on UPV was proposed. In general, the proportion of coarse aggregate in concrete structure is relatively large (up to approximately 40%), and its ultrasonic speed was large (up to approximately 6000 m/s). The coarse aggregate in the concrete was basically free from sulfate corrosion. If the influence of the coarse aggregate in the concrete is not eliminated, the change amount of the concrete ultrasonic pulse velocity value was directly used to evaluate the damage degree of sulfate corrosion in the concrete, and the results are often inaccurate. By calculating the proportion of coarse aggregate on a certain ultrasonic test line, the correction value of ultrasonic pulse velocity (UPV) of concrete structure was obtained. The effect of coarse aggregate on the concrete structure on the value of the UPV of the concrete specimen was eliminated, reflecting small changes in the value of the UPV of the concrete specimen caused by the cement matrix and pores so that the corrected change amount of the value of the UPV of the concrete could more correctly reflect the degree of corrosion damage in sulfate-attacked concrete;
(3) A method for evaluating the sulfate corrosion degree of concrete specimens based on AHP was constructed. By testing and correcting the UPV in multiple directions of the concrete sample and using the AHP method to calculate the weight and the overall relative corrosion degree makes the use of ultrasonic testing to judge changes in concrete structures under sulfate attack more in line with reality. The results of experimental and porosity changes showed that the overall relative corrosion evaluation results of concrete structures calculated by corrected UPV were more accurate.

## Figures and Tables

**Table 1:** Judgment matrix scale and its meaning.

$C_{jk}$ $\mathrm{or}\;(1-C_{jk})$	$V_{jk}$ $\mathrm{or}\;(V_{kj})$	Meaning
>0.99	1	The degree of corrosion in both directions is basically the same
0.95–0.99	2, 3	The degree of corrosion in one direction is a little bit more serious than the other
0.90–0.95	4, 5	The degree of corrosion in one direction is more serious than the other
0.75–0.90	6, 7	The degree of corrosion in one direction is quite a bit more serious than the other
≤0.75	8, 9	The degree of corrosion in one direction is extremely more serious than the other

**Table 2:** 3D judgment matrix of concrete corrosion degree.

Target Layer	$V_{x}$	$V_{y}$	$V_{z}$
$V_{x}$	$V_{11}=1$	$V_{12}$	$V_{13}$
$V_{y}$	$V_{21}$	$V_{22}=1$	$V_{23}$
$V_{z}$	$V_{31}$	$V_{32}$	$V_{33}=1$

**Table 3:** Random average consistency indexes.

*n*	1	2	3	4	5	6	7	8	9	10	11	12	13	14	15
RI	0.00	0.00	0.52	0.89	1.12	1.26	1.36	1.41	1.46	1.49	1.52	1.54	1.56	1.58	1.59

**Table 4:** The evaluation classification of the relative corrosion degree.

ADI	Relative Corrosion Coefficient	Classification
$C^{\prime }<0.85$		Extremely corrosion
$C^{\prime }\geq 0.85$	K≥1	No corrosion
	0.95<K<1	Mild corrosion
	0.9<K≤0.95	Medium corrosion
	0.8<K≤0.9	High corrosion
	K≤0.8	Extremely corrosion

**Table 5:** The UPV of the limestone (m/s).

Samples of Limestone	*z*-Direction	*x*-Direction	*y*-Direction	Average Value
1	5978.00	5960.00	6012.00	5983.33
2	5972.00	6065.00	5980.00	6005.67
Average value	5975.00	6012.50	5996.00	5994.50

**Table 6:** The proportion of coarse aggregate on the ultrasonic survey line.

Direction	The Length of Coarse Aggregate (mm)	The Proportion of Coarse Aggregate (ω)
*x*	16.79	33.58%
*y*	22.54	45.08%
*z*	40.13	40.13%

**Table 7:** Correction value of the UPV values after removing the effect of coarse aggregate (m/s).

Direction	The Original State ($V_{0}$)		The First Circle ($V_{1}$)		The Second Circle ($V_{2}$)	
	Corrected Value	Measuring Value	Corrected Value	Measuring Value	Corrected Value	Measuring Value
*x*	3068.8981	3670.4321	2963.2226	3569.3141	2908.3193	3516.2073
*y*	3159.5703	4015.6873	2956.5488	3832.0138	2832.7579	3716.4092
*z*	3085.3554	3831.5583	3009.0237	3760.6300	2924.8742	3681.3800

**Table 8:** The corrected and measuring UPV values of the specimen (m/s).

	The Original State		The First Circle		The Second Circle	
	Corrected Value	Measuring Value	Corrected Value	Measuring Value	Corrected Value	Measuring Value
UPV	3088.6018	3747.8150	2969.7133	3637.8654	2866.0668	3582.9285

**Table 9:** The relative corrosion coefficient of the sulfate attack of the specimen.

	The Original State		The First Circle		The Second Circle	
	Corrected Value	Measuring Value	Corrected Value	Measuring Value	Corrected Value	Measuring Value
Relative corrosion coefficient	1	1	0.9615	0.9707	0.9279	0.9560
Assessment Result			Mild corrosion	Mild corrosion	Medium corrosion	Mild corrosion

**Table 10:** Calculation results of overall porosity of concrete specimen.

	The Original State	The First Circle	The Second Circle
Porosity (%)	0.65	1.50	2.82

## Data Availability

The data that support the findings of this study are available upon request from the authors.
